# Adolescence risk factors for meniscus and ligamentous knee injuries in adulthood: A longitudinal study

**DOI:** 10.1002/ksa.12752

**Published:** 2025-07-13

**Authors:** Matias Vaajala, Alisa Teuho, Rasmus Liukkonen, Ville Ponkilainen, Arja Rimpelä, Leena K. Koivusilta, Ville M. Mattila

**Affiliations:** ^1^ Faculty of Medicine and Life Sciences University of Tampere Tampere Finland; ^2^ Department of Orthopaedics and Traumatology Tampere University Hospital Tampere Finland; ^3^ Unit of Health Sciences Faculty of Social Sciences Tampere University Tampere Finland; ^4^ Department of Adolescent Psychiatry Tampere University Hospital Tampere Finland; ^5^ Department of Social Research Faculty of Social Sciences University of Turku Turku Finland

**Keywords:** epidemiology, knee ligament injury, meniscus injury, risk factor

## Abstract

**Purpose:**

Our aim is to investigate the influence of adolescent health‐related behaviours on the occurrence of meniscus and ligament injuries in a large cohort of Finnish adolescents with an average 27‐year follow‐up.

**Methods:**

The baseline data were surveys conducted biannually from 1981 to 1997, and these were individually linked to outcomes, as well as meniscus and ligamentous knee injuries obtained from the Care Register for Health Care. A total of 47,747 participants were included. Of these, 22,020 were male and 25,727 were female. A Cox regression model was used to analyze the associations between exposure variables in adolescence (physical activity in sports clubs, other physical activity, overweight, smoking, monthly drunkenness, presence of chronic disease, family socioeconomic status) and the risk for knee injuries. Adjusted hazard ratios (aHR) with 95% confidence intervals (CIs) were computed.

**Results:**

The mean follow‐up time among the participants was 26.8 years (standard deviation [SD]: 4.1 years). A total of 1163 (2.4%) persons suffered a meniscus injury, and 1831 (3.8%) suffered a ligament injury. High physical activity in sports clubs (aHR: 2.02, CI: 1.85–2.21), overweight (aHR: 1.37, CI: 1.12–1.68), monthly drunkenness (aHR: 1.37, CI: 1.19–1.57) and presence of chronic disease (aHR 1.30, CI 1.08–1.56) increased the risk for meniscus injuries. Similarly, high (aHR: 1.72, CI: 1.60–1.84) physical activity in sports clubs, overweight (aHR: 1.26, CI 1.07–1.47), monthly drunkenness (aHR: 1.26, CI 1.13–1.41) and presence of chronic disease (aHR: 1.36, CI: 1.17–1.57) increased the risk for ligament injuries.

**Conclusions:**

Frequent physical activity in sports clubs presented a higher risk for meniscus and knee ligament injuries in individuals under 40, compared to degenerative factors such as being overweight or smoking. Interventions focusing on the suitable load level among adolescents with frequent and high‐intensity physical activity, in addition to efforts to reduce negative habits should be prioritized to lower the knee injury risk.

**Level of Evidence:**

Level II.

**Abbreviations**
ACL: anterior cruciate ligament
aHR: adjusted hazard ratio
CI: confidence interval
DAG: directed acyclic graph
SES: socioeconomic status

## BACKGROUND

Acute knee injuries are common traumas with high incidence in all age groups [33], accounting for up to 8% of cases in emergency departments [13]. The most common knee injuries are meniscal tears, affecting an estimated 12% of the adult population [11]. Among athletes, however, in certain sports such as football, the incidence of anterior cruciate ligament (ACL) tears is reported to have a truly high incidence [9], and a high reoccurrence rate among athletes [30]. Current understanding is that the aetiology behind meniscal tears can be degenerative, due to acute force to the knee, or a combination of these [35], while ligament injuries usually occur due to acute force to the knee, rather than degenerative changes in the adult population.

The continuum of meniscal tears varies from high‐energy traumatic knee injury of a young person with hemarthrosis to non‐traumatic degenerative meniscal tears associated with osteoarthrosis [46]. The main risk factors for degenerative meniscal tears are age over 60 years, male gender, and work‐related kneeling, squatting and climbing stairs [40]. A previous study has shown that female sex, prior reconstruction of the ACL, and familial predisposition were associated with isolated ACL injuries [38]. In a systematic review, female sex and specific measures of bony geometry of the knee joint were the highest risk factors for ACL injuries [38].

It is likely that among younger adults, these injuries are a result of a combination of long‐term load and stress on the ligaments of the knee caused by lifestyle factors, resulting in early degeneration, or a sudden force causes them in the healthy knee. In addition to injury risk related to physical activity, lower socioeconomic status (SES) [39], excessive alcohol consumption [8] and smoking in early life are known to be associated with injury risk through various mechanisms [17]. This study aims to investigate the influence of adolescent health‐related behaviours (frequent physical activity, overweight, drunkenness and smoking), self‐reported chronic disease and low family SES on the occurrence of meniscus and ligament injuries in a large cohort of Finnish adolescents with an average 27‐year follow‐up.

## METHODS

### Study design

This longitudinal study involved linking survey data from the Adolescent Health and Lifestyle Survey (AHLS) with sociodemographic data provided by Statistics Finland and outcome data obtained from the Care Register for Health Care (previously known as the Hospital Discharge Register) [21]. The follow‐up period for each participant concluded either at the first occurrence of a meniscus or ligamentous knee injury (diagnosed through clinical and/or radiological evaluation) or at the end of the study period on 31 December 2018.

### Baseline data

The baseline data were obtained from the AHLS. Initiated in 1977, surveys were conducted biennially and mailed to all Finnish individuals aged 14, 16 or 18, born on specific dates in June, July or August. The surveys were distributed between February and March, with follow‐ups for participants starting from April 30 of the survey year. Samples were selected from the Population Register Centre, and two reminder inquiries were sent to non‐respondents. This study used data collected between 1981 and 1997, including 47,747 participants (22,020 males and 25,727 females). The overall response rate was 75.1%.

### Outcome variable

**Figure 1:**
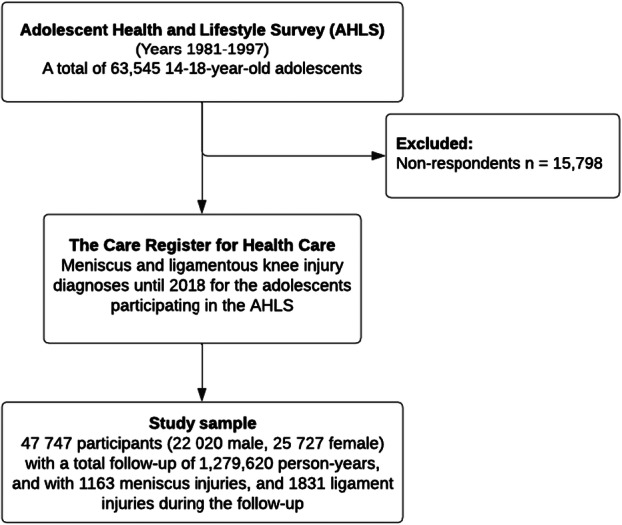
Flowchart depicting the formation of the study sample. Data from the Adolescent Health and Lifestyle Survey were linked with the data on knee injury‐related medical records found in the Care Register for Health Care.

The outcome measured was a hospitalization or outpatient visit related to meniscus or ligamentous knee injuries, with only the first diagnosis per patient being included. Outcome data were sourced from the Care Register for Health Care, which provides comprehensive information on participants treated in inpatient care, day surgeries and specialized outpatient care. The quality and coverage of this register are considered reliable. Diagnoses were identified using the International Classification of Diseases 10th revision (ICD‐10) for meniscus and ligamentous knee injuries, starting in 1998. The follow‐up began in 1998 because the ICD‐9 codes, which were used before 1997 and partly during that year, did not clearly distinguish between different types of knee injuries. Additionally, registry data quality improved significantly from 1998 onward, and the number of ICD‐9 classified injuries in our data set was very low [41]. Sensitivity analyses were conducted using surgeries for meniscus or ligament injuries as the outcome. Patients undergoing surgery were identified using NOMESCO (Nordic Medico‐Statistical Committee) operation codes (Table 1). The specific ICD‐10 and NOMESCO operation codes used in the study are detailed in Table 1, and the process for forming the final study sample is illustrated in Figure 1.

**Table 1:** The classification of hospitalizations due to meniscus injuries and ligamentous knee injuries according to ICD‐10 diagnostic codes and meniscus and cruciate ligament surgeries according to NOMESCO operation codes.

Meniscus injuries
**S83.2** Tear of meniscus, current injury
Ligamentous knee injuries
**S83.4** Sprain of collateral ligament of knee
**S83.5** Sprain of cruciate ligament of knee
**S83.6** Other or unspecified sprain of knee ligament
Meniscus surgeries
**NGD00** Partial excision of meniscus of knee, open
**NGD05** Partial excision of meniscus of knee, arthroscopic
**NGD10** Total excision of meniscus of knee, open
**NGD15** Total excision of meniscus of knee, arthroscopic
**NGD20** Reinsertion of meniscus of knee, open
**NGD25** Reinsertion of meniscus of knee, arthroscopic
**NGD50** Transposition of meniscus of knee
Cruciate ligament surgeries
**NGE30** Plastic repair of ligament of knee not using prosthetic material, anterior cruciate, open
**NGE35** Plastic repair of ligament of knee not using prosthetic material, anterior cruciate, arthroscopic
**NGE40** Plastic repair of ligament of knee not using prosthetic material, posterior or anterior cruciate, open
**NGE45** Plastic repair of ligament of knee not using prosthetic material, posterior or anterior cruciate, arthroscopic

### Explanatory variables

Health behaviour and chronic disease variables were sourced from the AHLS, while family SES variables were derived from the national registries of Statistics Finland. The presence of chronic disease was based on the following question. ‘Do you have a chronic disease or disability that disturbs your everyday life?’. Overweight was measured by body mass index (BMI), which was based on self‐reported height and weight. Overweight was defined using international age‐ and sex‐specific cut‐off points by Cole [10]. Table 2 provides a summary of the variables used in the analyses along with their original sources [44].

**Table 2:** Description of explanatory variables used in the analyses.

Variable and its source	Original variable and formation of the variable used in the analyses	Values
Family SES, Statistics Finland	Occupation‐based socioeconomic status of the respondent's mother and father from the national registries of Statistics Finland. The registry data on socioeconomic circumstances had been obtained from national censuses conducted every fifth year until 1995 and from on‐line registry data on a yearly basis from 2000 onwards. Classification of Statistics Finland.^18^	Both parents' unknown = 0 Both parents' upper white‐collar = 1 Either one upper white‐collar = 2 Either one lower white‐collar = 3 Either one blue‐collar = 4
Daily use of tobacco, AHLS	Combining questions on tobacco experimentation and frequency of tobacco use.	No = 1 Yes = 2
Monthly drunkenness, AHLS	Question on frequency of alcohol use and drunkenness.	No = 1 (Abstinence or only occasional drinking) Yes = 2 (Drunk once or more often a month)
Frequency of physical activity in sport clubs, AHLS	Frequency of participation in physical exercise in sports clubs.	Low = 1 (Once a week or less) Medium = 2 (2–3 times a week or less) High = 3 (4 or more times a week)
Frequency of other physical activity in leisure time, AHLS	Frequency of participation in physical exercise in leisure time.	Low = 1 (Once a week or less) Medium = 2 (2 to 3 times a week or less) High = 3 (4 or more times a week)
Self‐reported presence of chronic disease or disability AHLS	Question on long‐term disease or disability that disturbs your everyday life, which was based on the following question. ‘Do you have a chronic disease or disability that disturbs your everyday life?’	No = 1 Yes = 2
Overweight AHLS	BMI calculated from self‐reported height (cm) and weight (kg) and defined using international age‐ and sex‐specific cut‐off points by Cole.	No = 1 (Normal weight) Yes = 2 (High BMI according to Cole's criteria)

Abbreviations: AHLS, Adolescent Health and Lifestyle Survey; BMI, body mass index; SES, socioeconomic status.

### Statistical methods

Continuous variables were reported as means with standard deviations (SDs) (Table 3), while categorical variables were presented as absolute numbers and percentages. The Cox regression model was employed to assess risk factors for the main outcomes. The start of the follow‐up for each adolescent was the first of April each study year. The end‐point of the follow‐up was the occurrence of the first meniscus, death or ligamentous knee injury, or the end of the follow‐up, which was 31 December 2018. The proportional hazards assumption was tested using Schoenfeld residuals. Violations of proportional hazard assumptions were handled by constructing time‐stratified models [48]. Correlations of Schoenfeld residuals with time were repeatedly evaluated to ensure that the non‐proportionality was fixed. The explanatory variables included adolescent health and health behaviour variables: physical activity, high BMI, smoking, monthly drunkenness, presence of chronic disease and family SES. Physical activity in sports clubs and leisure time physical activity were analyzed separately. Adolescents with missing SES data for both parents were excluded from the SES models. Gender‐stratified models were also created. Additional analysis for meniscus and cruciate ligament surgeries is shown in Table S1 and only markable findings have been presented in the results. Also, sensitivity analyses using continuous BMI and a four‐level variable for alcohol use (0 = abstinent, 1 = occasional drinking, 2 = recurrent drinking [drunk more than once a month but rarely drunkenness], 3 = recurring drunkenness [weekly]) were performed due to the broad categorization used in the main analyses. Adjusted hazard ratios (aHR) with 95% confidence intervals (CIs) were compared between groups.

**Table 3:** Descriptive statistics on variables used in the study by gender.

	All		Male		Female	
	47,747		22,020		25,727	
Total number	*n*	%	*n*	%	*n*	%
Background information						
Age during the survey						
14 years	15,880	33.3	7533	34.2	8347	32.5
16 years	15,885	33.3	7320	33.3	8565	33.3
18 years	15,982	33.5	7167	32.6	8815	34.3
Age at the end of the follow‐up (years) (mean; SD)	42.7 (4.3)		42.7 (4.3)		42.7 (4.3)	
Family SES at age 15						
Both upper white‐collar	7775	16.3	3677	16.7	4098	16.0
Either one upper white‐collar	11,899	24.9	5456	24.8	6443	25.1
Either one lower white‐collar	15,184	31.8	7093	32.2	8091	31.5
Either on blue‐collar	10,423	21.8	4647	21.1	5776	22.5
Both unknown	2466	5.2	1147	5.2	1319	5.1
Explanatory variables						
Smoking in adolescence	11,564	24.2	5690	25.9	5874	22.8
Monthly drunkenness	9048	19.0	4892	22.2	4156	16.2
Physical activity in sports club						
High	4130	8.6	2751	12.5	1379	5.4
Medium	12,397	26.0	6168	28.0	6229	24.2
Low	20,058	42.0	8088	36.7	11,970	46.5
Unknown	11 162	23.4	5013	22.8	6149	23.9
Physical activity in leisure time						
High	9036	18.9	4531	20.6	4505	17.5
Medium	26,073	54.6	11,652	52.9	14,421	56.1
Low	1770	3.7	966	4.4	804	3.1
Unknown	10,868	22.8	4871	22.1	5997	23.3
High BMI in adolescence	4839	10.1	2838	12.9	2001	7.8
Chronic disease in adolescence	4222	8.8	1819	8.2	2403	9.3
Outcome variables in follow‐up						
Meniscus injury diagnosis	1163	2.4	789	3.6	374	1.5
Ligament injury diagnosis	1831	3.8	1162	5.3	669	2.6
Meniscus surgery	1561	3.2	1047	4.8	514	2.0
Cruciate ligament surgery	918	1.9	617	2.8	301	1.2

Abbreviations: BMI, body mass index; SD, standard deviation; SES, socioeconomic status.

Cox regression models were adjusted by selecting variables for a multivariable model based on directed acyclic graphs (DAGs). These variables were chosen according to established risk factors and hypothesized causal pathways. The DAGs were constructed using the free online software DAGitty (dagitty.net) (Figures [Link], [Link], [Link], [Link], [Link], [Link]) [42]. DAGitty automatically identifies potential sets of adjustment variables that could impact the main outcome. It determines the minimal adjustment set required to block all non‐causal paths, ensuring that no variable in the set is a descendant of the explanatory variable and that all backdoor paths are blocked when the set is conditioned upon. By utilizing algorithms from graph theory and causal inference, DAGitty streamlines this process and offers real‐time feedback on the adequacy of the selected adjustments, helping researchers make valid causal inferences [6]. In the DAGs, the exposure variable is positioned in the bottom left corner, while the outcome variable is placed in the right corner. Variables are colour‐coded based on their relationships: yellow indicates an ancestor of the exposure (influencing the exposure variable), blue represents an ancestor of the outcome (influencing the outcome variable), and red signifies an ancestor of both the exposure and the outcome variables. Pathways connecting variables are represented in green, red or black. A green pathway indicates a causal relationship between the exposure and the outcome variables. A red pathway represents a biasing relationship, while a black pathway connects the outcome to variables that influence only the outcome.

According to the DAGs, models with physical activity, smoking, monthly drunkenness or presence of chronic disease as the exposure variable were adjusted for the adolescent's age and family SES during adolescence. The model with BMI as the exposure variable was adjusted for age at the survey year, physical activity and family SES during adolescence. The model with family SES as the exposure variable was adjusted for the adolescent's age and smoking status during adolescence.

Statistical analyses were conducted using R version 4.0.5 (R Foundation for Statistical Computing) [7]. The results of this study are reported according to the STROBE (Strengthening the Reporting of Observational Studies in Epidemiology) guidelines [34].

## RESULTS

The mean follow‐up time among the AHLS participants was 26.8 years (SD: 4.1 years). A total of 1163 (2.4%) persons suffered a meniscus injury, and a total of 1831 (3.8%) suffered a ligament injury. All injuries were more common among males than females. The mean age at the time of the meniscus injury was 32.6 years (SD: 7.6 years), ligament injury 30.9 years (SD: 7.3 years) and cruciate ligament surgery 31.3 years (SD: 7.1 years). The most common first ligament injury diagnosis was sprain of cruciate ligament of knee (*n* = 917, 50.1%), followed by other or unspecified sprain of knee ligament (*n* = 667, 36.4%) (Tables 3 and 4).

**Table 4:** A total number of different first ICD‐10 diagnoses and NOMESCO operation codes among patients included in the study.

Meniscus injuries	*n*
**S83.2** Tear of meniscus, current injury	1163
Ligament injuries	*n*
**S83.4** Sprain of collateral ligament of knee	247
**S83.5** Sprain of cruciate ligament of knee	917
**S83.6** Other or unspecified sprain of knee ligament	667
Meniscus surgeries	
**NGD00** Partial excision of meniscus of knee, open	11
**NGD05** Partial excision of meniscus of knee, arthroscopic	1446
**NGD10** Total excision of meniscus of knee, open	9
**NGD15** Total excision of meniscus of knee, arthroscopic	47
**NGD20** Reinsertion of meniscus of knee, open	28
**NGD25** Reinsertion of meniscus of knee, arthroscopic	7
**NGD50** Transposition of meniscus of knee	13
Cruciate ligament injuries	*n*
**NGE30** Plastic repair of ligament of knee not using prosthetic material, anterior cruciate, open	32
**NGE35** Plastic repair of ligament of knee not using prosthetic material, anterior cruciate, arthroscopic	865
**NGE40** Plastic repair of ligament of knee not using prosthetic material, posterior or anterior cruciate, open	<5^a^
**NGE45** Plastic repair of ligament of knee not using prosthetic material, posterior or anterior cruciate, arthroscopic	19

*Note*: The first meniscus, ligamentous knee injury and operation were included, and the first diagnosed code for the hospitalization is shown in the table.

Abbreviation: ICD‐10, International Classification of Diseases 10th revision.

a Finnish legislation prevents reporting the exact rate if the value is lower than 5.

In the Cox regression analysis, high frequency physical activity in sports clubs markedly increased the risk for meniscus injuries (aHR: 2.02, CI: 1.85–2.21) and for knee ligament injuries (aHR: 1.72, CI: 1.60–1.83), when compared to adolescents with low frequent physical activity in sports clubs. This increased risk was evident in both genders, but was higher among females, increasing up to 1.95 (CI: 1.65–2.29) for meniscus injuries, and up to 1.89 (CI: 1.67–2.13) for ligament injuries. High frequency of other physical activity in leisure time showed no such increase for meniscus and knee ligament injuries when compared to those with low frequent physical activity, and no evidence of any difference according to the activity levels in leisure time was found among females (Table 5).

**Table 5:** Overall and gender‐stratified adjusted hazard ratios (aHRs) with 95% confidence intervals (CIs) for meniscus injuries and ligament injuries.

	All participants				Male				Female			
	Meniscus injury		Ligament injury		Meniscus injury		Ligament injury		Meniscus injury		Ligament injury	
	aHR	CI	aHR	CI	aHR	CI	aHR	CI	aHR	CI	aHR	CI
Physical activity in sports club^a^												
Low	1.00		1.00		1.00		1.00		1.00		1.00	
Medium	1.74	1.48–2.04	1.57	1.39–1.77	1.90	1.55–2.32	1.65	1.41–1.92	1.21	0.92–1.60	1.24	1.02–1.52
High	2.02	1.85–2.21	1.72	1.60–1.84	1.84	1.65–2.05	1.49	1.37–1.63	1.95	1.65–2.29	1.89	1.67–2.13
Physical activity in leisure time^a^												
Low	1.00		1.00		1.00		1.00		1.00		1.00	
Medium	1.12	0.78–1.60	1.28	0.97–1.72	1.42	0.91–2.20	1.82	1.23–2.70	0.90	0.49–1.66	0.87	0.57–1.34
High	1.25	1.04–1.50	1.31	1.13–1.52	1.42	1.13–1.78	1.56	1.27–1.91	1.01	0.73–1.39	1.03	0.82–1.29
BMI^b^												
Normal BMI	1.00		1.00		1.00		1.00		1.00		1.00	
High BMI	1.37	1.12–1.68	1.26	1.07–1.47	1.14	0.89–1.44	1.12	0.93–1.36	1.49	1.01–2.19	1.21	0.89–1.63
Tobacco use^a^												
No smoking	1.00		1.00		1.00		1.00		1.00		1.00	
Smoking	1.06	0.93–1.21	1.09	0.98–1.21	0.95	0.80–1.11	1.00	0.88–1.15	1.19	0.94–1.50	1.16	0.98–1.39
Monthly drunkenness^a^												
Abstinence or occasional	1.00		1.00		1.00		1.00		1.00		1.00	
At least monthly	1.37	1.19–1.57	1.26	1.13–1.41	1.32	1.12–1.54	1.27	1.11–1.45	1.12	0.85–1.46	1.03	0.84–1.26
Chronic disease^a^												
No	1.00		1.00		1.00		1.00		1.00		1.00	
Yes	1.30	1.08–1.56	1.36	1.17–1.57	1.21	0.96–1.53	1.29	1.07–1.56	1.58	1.17–2.12	1.54	1.23–1.92
Family socioeconomic status^c^												
Both parents upper white‐collar	1.00		1.00		1.00		1.00		1.00		1.00	
Either one upper white‐collar	1.16	0.96–1.40	1.15	0.98–1.34	1.00	0.80–1.25	1.19	0.98–1.44	1.79	1.22–2.62	1.10	0.85–1.42
Either one lower white‐collar	1.07	0.97–1.17	1.07	1.00–1.15	1.00	0.90–1.11	1.09	0.99–1.19	1.31	1.09–1.58	1.04	0.92–1.18
Either one blue‐collar	0.99	0.89–1.10	0.98	0.90–1.06	0.91	0.79–1.03	0.96	0.86–1.07	1.22	1.01–1.47	1.01	0.87–1.15

Abbreviation: BMI, body mass index.

^a^ Adjusted by the age at the end of the follow‐up and family socioeconomic status in adolescence.

^b^ Adjusted by the age at the end of the follow‐up, physical activity and family socioeconomic status in adolescence.

^c^ Adjusted by the age at the end of the follow‐up and smoking status in adolescence.

When compared to adolescents with normal BMI, high BMI increased the risk for meniscus injuries (aHR: 1.37, CI: 1.12–1.68) and ligament injuries (aHR: 1.31, CI: 1.13–1.52). No evidence of a difference was found in gender‐stratified models. Also, no evidence of a difference between daily smokers and non‐smokers in the meniscus or knee ligament injuries was found. Monthly drunkenness in adolescence increased the risk for meniscus injuries (aHR: 1.37, CI: 1.19–1.57) and for ligament injuries (aHR: 1.26, CI: 1.13–1.41) when compared to adolescents who were abstinent or occasional drinkers. These risks were higher among males (aHR: 1.32, CI: 1.12–1.54 for meniscus injuries, and aHR: 1.27, CI: 1.11–1.45 for ligament injuries), and no evidence of an impact was found among females. Presence of chronic disease in adolescence increased the risk for meniscus injuries (aHR: 1.30, CI: 1.08–1.56) and for ligament injuries (aHR: 1.36, CI: 1.17–1.57), when compared to adolescents without chronic diseases. This risk was higher among females (aHR: 1.58, CI 1.17–2.12 for meniscus injuries, and aHR: 1.54, CI: 1.23–1.92 for ligament injuries), and no evidence of difference was found among males (Table 5). When compared to the findings on the meniscus and ligament injuries, the additional sensitivity analysis of knee surgeries, and for alcohol use and BMI, showed similar results to the main analyses. We found that even occasional drinking in adolescence increased the risk for ligamentous knee injuries in adulthood (aHR: 1.20, CI: 1.08–1.33) (Tables S1 and S2).

## DISCUSSION

The main finding of this study was that high frequent physical activity in sports clubs was a more important risk factor for meniscus and ligament injury of knee than degenerative background variables, such as overweight and smoking before the age of 40, indicating that meniscal and ligamentous injuries in this age group are more due to trauma than degeneration. This increased risk was evident in both genders, but was higher among females. Other physical activity in leisure time served as a small risk factor for meniscus and ligament injuries among males, but the risk was small compared to the risk caused by physical activity in sports clubs. In addition, no evidence of difference in the risk for ligament or meniscus injuries was found among females with high other physical activity. In addition, being overweight increased the risk for meniscus and ligamentous knee injuries in the model for all participants, but no difference was found in gender‐stratified models. Also, monthly drunkenness increased the risk among males, and the presence of chronic disease increased the risk, especially among females. We found no evidence of a different risk for meniscus or ligament injuries between smokers and non‐smokers.

High‐frequency physical activity in sports clubs in adolescence increased the risk for meniscus injuries and ligamentous knee injuries in adulthood, as expected. These are usually caused by high‐impact traumas occurring in many sports [12, 28]. The risk of meniscus and ligament injuries was higher among females. According to a large sports injury surveillance, the highest rates of recurrent ACL ruptures were among male football players, female gymnasts and female soccer players [14]. Similar findings have also been found in other studies [9]. In addition, meniscal tears are common in sports [1]. Females have a higher risk than males for knee injuries, which is consistent with the findings in previous literature. Especially among athletes, females have consistently been reported to have a higher risk for ACL injuries [5, 9]. Studies on youth sports participation in Finland show that from 1985 to 2014, the proportion of 11‐year‐olds in organized sports clubs increased, with a notable rise, especially among girls [27]. However, gender differences in sports preferences persist—boys tend to engage more in team sports like football, floorball and ice hockey, while girls favour activities such as dance and gymnastics, which may contribute to variations in injury risk, as girls often participate in activities with high‐impact landings and repetitive stress explaining the higher injury rates observed in our study [15]. In addition, differences in muscle strength development between male and female athletes, particularly the slower increase in knee extensor strength and the decline in relative knee flexor strength in adolescent girls during maturation, may contribute to a higher injury risk among females in early adulthood [32].

Interestingly, among females, in contrast to the higher risk in physical activity in sports clubs, females with a higher frequency of physical activity in leisure time had no increased risk for meniscus and knee ligament injuries. This might be explained by the lower intensity and flexibility of leisure time activities, whereas adolescents with high physical activity in sports clubs most likely are more competitive and put their joints at higher risk for injuries. All physical activity in adolescence supports the growth of the bone and muscle tissues, leading to more stable and powerful lower limbs [22]. It is good to note that physical activity in adolescence might differ from adolescence to adulthood. It is known that physical activity decreases from adolescence to adulthood, even when high physical activity levels are maintained in adolescence [2, 45]. Performing high‐level sports in adulthood usually requires high physical activity already in adolescence, which is why these persons are at the highest risk for acute knee injuries, such as meniscal tears and ACL ruptures [37].

Monthly drunkenness in adolescence was a risk factor for meniscus and knee ligament injuries among males, but not among females. Different drinking habits between the genders likely explain the difference. Finnish males consume alcohol more often and larger amounts than females [24], and experience and cause more alcohol‐related injuries than females, which could contribute to injury risk also in the knee area [24, 26, 47]. In addition, excessive alcohol consumption increases the risk for knee osteoarthritis, but no association between chronic alcohol consumption and ligamentous knee injuries has been reported [23]. When interpreting the results, it should be acknowledged that at this age, the use of alcohol is not the norm and drunkenness even less. Adolescents are not allowed to have access to alcohol legally before age 18. Most 14‐year‐olds use alcohol rarely or not at all. When we follow these adolescents for a long period, the use of alcohol or drunkenness is hardly ever an immediate cause of knee injury, but most often, there is a longer time between the explanatory variable and the outcome. Alcohol use at a young age is described here more as a lifestyle in later life; it is known that alcohol use predicts later alcohol use [4]. Interestingly, the presence of self‐reported chronic diseases increased the risk for meniscus injuries and ligamentous knee injuries among females. As we have no information on the types of chronic diseases, just on the presence of these, the reasons in the aetiology behind the increased risk remain unknown and are most likely multidimensional. Chronic diseases might decrease the quality of life and therefore lead to more challenging adulthood, with increased risk for traumatic events through various routes. No previous studies have reported a higher risk for acute knee injuries among younger persons with chronic diseases. Furthermore, self‐reported variables are always prone to bias, and this should be considered when interpreting the results. A previous study investigating the self‐reports of chronic diseases has reported a small number of inconsistencies [20].

In addition to high physical activity, being overweight increased the risk for meniscus tears and ligamentous knee injuries for all participants, but no evidence of difference was found in gender‐stratified models. In previous literature, overweight has been a risk factor for ligamentous knee injuries among both genders [3, 19]. Obesity is also known to increase the risk of fractures in the lower limb [43]. However, the literature on this topic is limited to a few studies. Being overweight increases the load on the joints in the lower limb, which contributes to the degenerative processes in the knee [49], possibly increasing the risk for meniscus injuries and ligamentous injuries. In our study, overweight was not related to knee injuries in males or in females, which was an unexpected finding. Further, we found no evidence of an association between smoking status and the risk for knee injuries. Smoking is known to be associated with increased cartilage loss [36], and smoking negatively affects the healing process after ligamentous knee injury or knee cartilage surgeries [16, 29], but no study has investigated the effects of smoking on the ligamentous knee injury risk. Based on our results, smoking in adolescence plays no role in the occurrence of meniscus and knee ligament injuries, which is relatively small.

We hypothesized that knee injuries can be a result of a combination of long‐term load and stress caused by lifestyle factors resulting in early degeneration or a result of a sudden force to the healthy knee caused by high‐intensity sports. The results of our study are in line with the hypothesis. As expected, adolescents with frequent physical activity in sports clubs had a notably higher risk for knee injuries. However, it appears that non‐competitive physical activity does not increase the risk for knee injuries as much as previously thought in previous literature. These results suggest that, especially in sports clubs, adopting a multifaceted approach that combines individual training programmes with general injury‐prevention strategies is beneficial. These should include personalized strength‐training programmes, neuromuscular training, load management and comprehensive warm‐up programmes, and increasing injury awareness among adolescents. In addition, focusing interventions on the suitable load level in terms of knee health and prevention of health‐damaging behaviours, such as smoking, being overweight and alcohol use, should be performed in both genders in terms of knee injuries, too.

The findings of this study should be interpreted in the context of Finland's healthcare system characteristics. Finland has a well‐established public healthcare system that provides equal access to medical care [18]. The validity of the Finnish healthcare registries is also good [41]. In countries with private insurance, access to healthcare may be limited for certain individuals. Differences in physical activity, injury prevention programmes or healthcare access in other countries may limit the generalizability of these results. On the other hand, the use of alcohol and smoking reflects adolescent risky behaviours, which is why it is likely that these results can be generalized worldwide. Future studies should examine whether similar associations are observed in diverse healthcare and sports environments.

A key strength of this study is its long follow‐up period, averaging 27 years, which provides insights into how adolescent risk factors may influence adult ligamentous knee injuries in mid‐adulthood. The nationwide sample was large and representative of Finnish adolescents. However, the study has some limitations. All health behaviour and chronic disease data were self‐reported, which may introduce inaccuracies and bias, such as younger adolescents potentially underreporting smoking or alcohol use out of concern that their parents might see their responses. Due to the nature of the data and the original variable definitions, we had to rely on a very coarse categorization (e.g., alcohol use and BMI), as a more detailed analysis was not feasible given the limitations of self‐reported data and the broad, subjective classifications in the original data set. However, the performed sensitivity analyses using more specific variables for alcohol use and BMI showed similar results to our main analyses, highlighting that even restrained use of alcohol in adolescence increased the risk for ligamentous knee injuries. On the other hand, the reliability and validity of self‐reported smoking status are generally considered good among young adults [25]. Additionally, we cannot account for how health and health behaviours may have changed over time, although these factors, particularly smoking, are well‐documented to remain strongly correlated from adolescence to adulthood [31]. In addition, the diagnoses are not based on imaging and therefore are not absolutely precise. However, as a sensitivity analysis, we analyzed meniscus and ligament surgeries, which showed no significant differences from the main findings of this study, indicating that the reliability of the diagnoses included in this study is sufficient.

## CONCLUSIONS

Frequent physical activity in sports clubs posed a greater risk for meniscus and knee ligament injuries than degenerative factors, such as being overweight or smoking, for individuals under the age of 40, indicating that meniscal and ligamentous injuries in this age group are more due to trauma than degeneration. Interventions focusing on the suitable load level among adolescents with frequent and high‐intensity physical activity, in addition to efforts to reduce health‐damaging behaviours like smoking, drunkenness and being overweight, should be prioritized in both genders to lower the knee injury risk.

## AUTHOR CONTRIBUTIONS

All authors conceived and planned the project. Matias Vaajala wrote the initial manuscript. Matias Vaajala, Rasmus Liukkonen and Alisa Teuho analyzed and interpreted the data. Ville Ponkilainen provided support in statistical analyses and methodology. Leena Koivusilta and Arja Rimpelä played a major role in collecting the original data set. Ville Ponkilainen and Ville Mattila provided clinical expertise. Ville Mattila, Leena Koivusilta and Arja RImpelä supervised the study. All authors read and approved the final manuscript.

## CONFLICT OF INTEREST STATEMENT

The authors declare no conflicts of interest.

## ETHICS STATEMENT

The Finnish Social and Health Data Permit Authority granted the permit to use social and healthcare data (https://findata.fi) and linked that data with the data set of the Adolescent Health and Lifestyle Survey, measured biennially from 1981 to 1997. Statistics Finland linked these data sets. The study protocol was approved by its Institutional Review Board and by the Data Protection Ombudsman. Identification of the study participants was withheld from the investigators at all stages of the study, and the rights and duties of both parties were specified in the contract. The Joint Commission on Ethics of the University of Turku and the Turku University Hospital stated that no human rights were violated in the research protocol and approved it. Parental consent was neither considered by the ethics review boards at that time nor was it needed for linking the data sets. When the permission to use registered data was granted, the authorities were obliged to guarantee that the conditions described in the Personal Data Protection Act (523/1999) were followed (https://www.finlex.fi/fi/laki/alkup/1999/19990523). The Adolescent Health and Lifestyle Survey data were gathered from 1981 to 1997, at a time when there was no specific legislation or national guidelines on parental consent. The first review boards at the universities were established in Finland in the 1980s. AHLS was reviewed by the Ethical Review Board of the University of Helsinki, Department of Public Health in 1986, but parental consent was not considered at that time. The purpose of the study was stated on the first page of the questionnaire or in a separate information letter. Participants' informed consent was shown by their answers to the survey. The questionnaires did not include questions that involve a risk of causing mental harm that exceeds the limits of normal daily life to the research participants. It was stated that if parents would like to get to know the questionnaire, they were advised to do so before the adolescent answered. In later surveys of the Adolescent Health and Lifestyle Survey, the review boards waived parental consent, which is in line with the present national guidelines on ethical review in human sciences by the Finnish National Board on Research Integrity (https://tenk.fi/en/ethical-review/ethical-review-human-sciences).

## Supporting information

supmat.

Figure S1. NTTT polvi.

Figure S2. NTTT polvi.

Figure S3. NTTT polvi.

Figure S4. NTTT polvi.

Figure S5. NTTT polvi.

Figure S6. NTTT polvi.

Table S1. NTTT polvi.

Table S2. Knee lig.

## Data Availability

The data sets are available from the corresponding authors upon a reasonable request.
