# Supplementary material for: Adolescence risk factors for meniscus and ligamentous knee injuries in adulthood: A longitudinal study
Source: Knee Surg Sports Traumatol Arthrosc. 2025 Jul 13;34(4):1245–56. doi: 10.1002/ksa.12752 (PMC13037346; doi:10.1002/ksa.12752)
Supplement: Supplementary file 1 — supmat. [file KSA-34-1245-s001.docx]

**Supplementary table 1**: Additional analysis for knee surgeries. Adjusted hazard ratios (aHR) with 95% confidence intervals (CI) for meniscus surgeries and cruciate ligament surgeries.

**Supplementary table 2**. Sensitivity analysis for continuous BMI, and four-level variable for alcohol use. Overall and gender-stratified adjusted hazard ratios (aHR) with 95% confidence intervals (CI) for meniscus injuries and ligament injuries.

Supplementary Figure 1. DAG: Physical activity and the risk for meniscus or ligamentous knee

injuries.

Supplementary Figure 2. DAG: Higher BMI and the risk for meniscus or ligamentous knee injuries.

Supplementary Figure 3. DAG: Smoking and the risk for meniscus or ligamentous knee injuries.

Supplementary Figure 4. DAG: Monthly drunkenness and the risk for meniscus or ligamentous knee

injuries.

Supplementary Figure 5. DAG: Chronic diseases and the risk for meniscus or ligamentous knee

injuries.
