# Supplementary figures and images for: Adolescence risk factors for meniscus and ligamentous knee injuries in adulthood: A longitudinal study
Source: Knee Surg Sports Traumatol Arthrosc. 2025 Jul 13;34(4):1245–56. doi: 10.1002/ksa.12752 (PMC13037346; doi:10.1002/ksa.12752)

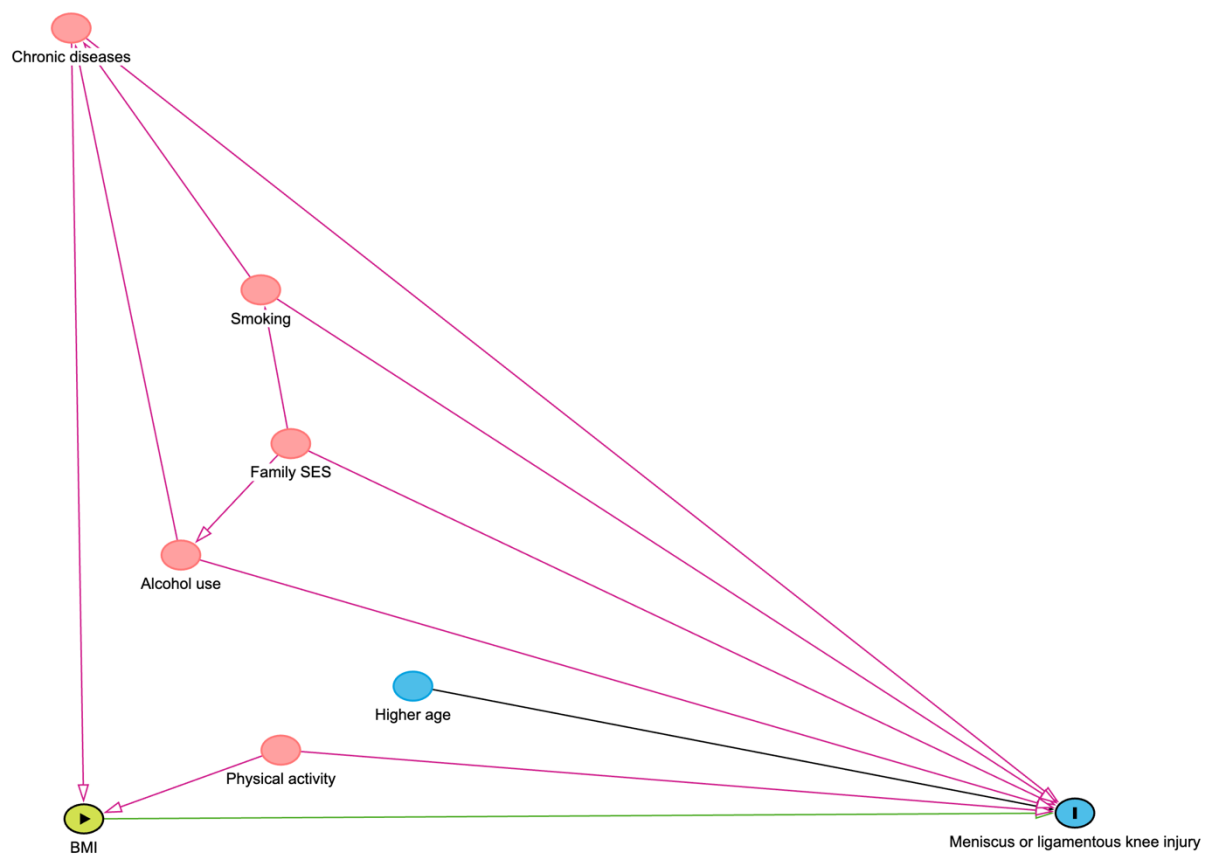

Supplementary Figure 2. DAG: Higher BMI and the risk for meniscus or ligamentous knee injuries.

Supplement: Supplementary file 3 — Figure S2. NTTT polvi. [file KSA-34-1245-s002.pdf]

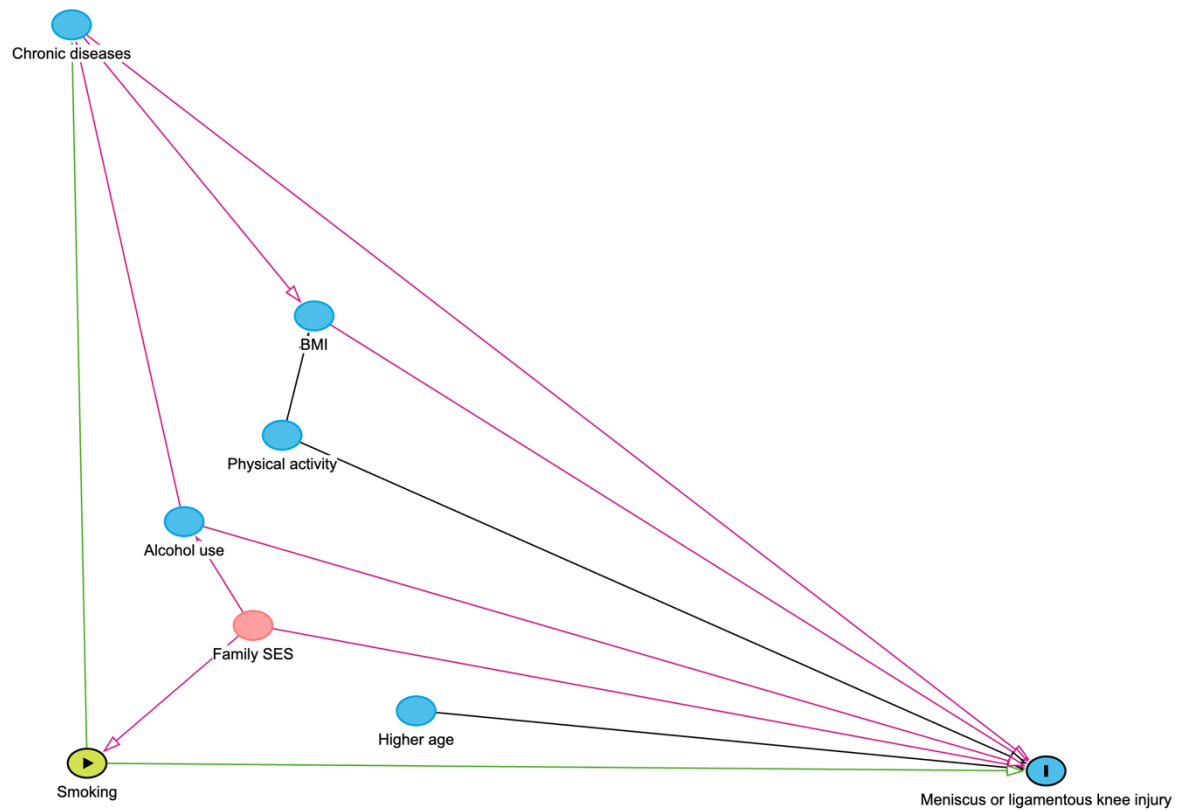

Supplementary Figure 3. DAG: Smoking and the risk for meniscus or ligamentous knee injuries.

Supplement: Supplementary file 4 — Figure S3. NTTT polvi. [file KSA-34-1245-s008.pdf]
