# Supplementary material for: Adolescence risk factors for meniscus and ligamentous knee injuries in adulthood: A longitudinal study
Source: Knee Surg Sports Traumatol Arthrosc. 2025 Jul 13;34(4):1245–56. doi: 10.1002/ksa.12752 (PMC13037346; doi:10.1002/ksa.12752)
Supplement: Supplementary file 6 — Figure S5. NTTT polvi. [file KSA-34-1245-s005.pdf]

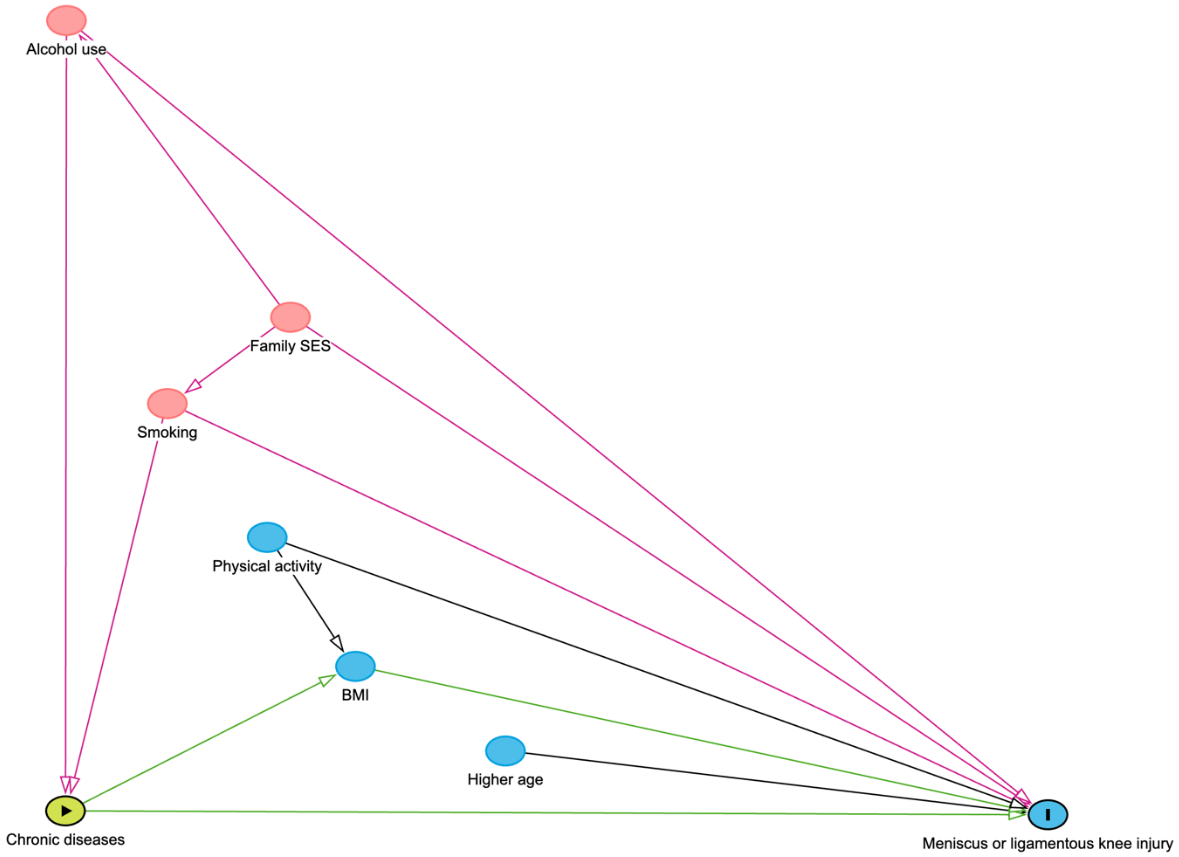

Supplementary Figure 5. DAG: Chronic diseases and the risk for meniscus or ligamentous knee injuries.
