# Supplementary material for: Adolescence risk factors for meniscus and ligamentous knee injuries in adulthood: A longitudinal study
Source: Knee Surg Sports Traumatol Arthrosc. 2025 Jul 13;34(4):1245–56. doi: 10.1002/ksa.12752 (PMC13037346; doi:10.1002/ksa.12752)
Supplement: Supplementary file 8 — Table S1. NTTT polvi. [file KSA-34-1245-s007.docx]

Supplementary table 1: Additional analysis for knee surgeries. Adjusted hazard ratios (aHR) with 95% confidence intervals (CI) for meniscus surgeries and cruciate ligament surgeries.

|  | Meniscus surgery | | Cruciate ligament surgery | |
| --- | --- | --- | --- | --- |
|  | aHR | CI | aHR | CI |
| Physical activity in sports club^a^ |  |  |  |  |
| low | 1.00 |  | 1.00 |  |
| medium | 1.53 | 1.33-1.77 | 2.06 | 1.73-2.48 |
| high | 1.79 | 1.65-1.94 | 1.91 | 1.73-2.12 |
| Physical activity in leisure time^a^ |  |  |  |  |
| low | 1.00 |  | 1.00 |  |
| medium | 0.99 | 0.73-1.34 | 1.26 | 0.84-1.89 |
| high | 1.11 | 0.95-1.29 | 1.26 | 1.03-1.56 |
| BMI^b^ |  |  |  |  |
| normal BMI | 1.00 |  | 1.00 |  |
| high BMI | 1.58 | 1.33-1.88 | 1.15 | 0.91-1.46 |
| Tobacco use^a^ |  |  |  |  |
| no smoking | 1.00 |  | 1.00 |  |
| smoking | 1.15 | 1.03-1.29 | 1.04 | 0.89-1.21 |
| Monthly drunkenness^a^ |  |  |  |  |
| abstinence or occasional | 1.00 |  | 1.00 |  |
| drunk once or more a month | 1.32 | 1.17-1.49 | 1.27 | 1.08-1.48 |
| Chronic disease^a^ |  |  |  |  |
| no | 1.00 |  | 1.00 |  |
| yes | 1.29 | 1.10-1.51 | 0.98 | 0.78-1.23 |
| Family socioeconomic status^c^ |  |  |  |  |
| Both parents upper white-collar | 1.00 |  | 1.00 |  |
| Either one upper white-collar | 1.09 | 0.92-1.29 | 1.15 | 0.93-1.43 |
| Either one lower white-collar | 1.10 | 1.02-1.19 | 1.09 | 0.98-1.21 |
| Either one blue-collar | 1.04 | 0.95-1.30 | 0.98 | 0.87-1.11 |

^a^ Adjusted by the age at the end of the follow-up and family socioeconomic status in adolescence

^b^ Adjusted by the age at the end of the follow-up, physical activity, and family socioeconomic status in adolescence

^c^ Adjusted by the age at the end of the follow-up, and smoking status in adolescence
