# Supplementary material for: Adolescence risk factors for meniscus and ligamentous knee injuries in adulthood: A longitudinal study
Source: Knee Surg Sports Traumatol Arthrosc. 2025 Jul 13;34(4):1245–56. doi: 10.1002/ksa.12752 (PMC13037346; doi:10.1002/ksa.12752)
Supplement: Supplementary file 9 — Table S2. Knee lig. [file KSA-34-1245-s004.docx]

**Supplementary table 2**. Sensitivity analysis for continuous BMI, and four-level variable for alcohol use. Overall and gender-stratified adjusted hazard ratios (aHR) with 95% confidence intervals (CI) for meniscus injuries and ligament injuries.

|  | All participants | | | | Male | | | | Female | | | |
| --- | --- | --- | --- | --- | --- | --- | --- | --- | --- | --- | --- | --- |
|  | Meniscus injury | | Ligament injury | | Meniscus injury | | Ligament injury | | Meniscus injury | | Ligament injury | |
|  | aHR | CI | aHR | CI | aHR | CI | aHR | CI | aHR | CI | aHR | CI |
| BMI^a^ |  |  |  |  |  |  |  |  |  |  |  |  |
| Risk increase per BMI-unit | 1.03 | 1.01-1.05 | 1.03 | 1.01-1.04 | 1.02 | 1.00-1.04 | 1.01 | 0.99-1.03 | 1.02 | 0.99-1.06 | 1.05 | 1.02-1.07 |
| Monthly drunkenness^b^ |  |  |  |  |  |  |  |  |  |  |  |  |
| abstinence | 1.00 |  | 1.00 |  | 1.00 |  | 1.00 |  | 1.00 |  | 1.00 |  |
| occasional drinking | 1.16 | 1.01-1.32 | 1.20 | 1.08-1.33 | 1.25 | 1.06-1.48 | 1.19 | 1.04-1.37 | 1.09 | 0.87-1.37 | 1.31 | 1.11-1.55 |
| recurrent drinking | 1.21 | 1.11-1.31 | 1.31 | 1.11-1.55 | 1.22 | 1.11-1.35 | 1.16 | 1.09-1.24 | 1.06 | 0.91-1.24 | 1.19 | 1.10-1.28 |
| recurring drunkenness | 1.12 | 1.02-1.23 | 1.14 | 1.06-1.23 | 1.08 | 0.97-1.20 | 1.09 | 1.00-1.19 | 1.08 | 0.90-1.31 | 1.13 | 0.98-1.30 |

^a^ Adjusted by the age at the end of the follow-up, physical activity, and family socioeconomic status in adolescence

^b^ Adjusted by the age at the end of the follow-up and family socioeconomic status in adolescence
